# Validation and Use of an Accurate, Sensitive Method for Sample Preparation and Gas Chromatography–Mass Spectrometry Determination of Different Endocrine-Disrupting Chemicals in Dairy Products

**DOI:** 10.3390/foods10051040

**Published:** 2021-05-10

**Authors:** Laura Palacios Colón, Andrés J. Rascón, Lamia Hejji, Abdelmonaim Azzouz, Evaristo Ballesteros

**Affiliations:** 1 Department of Physical and Analytical Chemistry, E.P.S of Linares, University of Jaén, 23700 Linares, Spain; lpcolon@ujaen.es (L.P.C.); ajrl0006@red.ujaen.es (A.J.R.); lamiae.hejji@gmail.com (L.H.); aazzouz@uae.ac.ma (A.A.); 2 Department of Chemistry, Faculty of Science, University of Abdelmalek Essaadi, B.P. 2121, M’Hannech II, Tétouan 93002, Morocco

**Keywords:** milk, dairy products, endocrine disrupting chemicals, solid-phase extraction, gas chromatography–mass spectrometry

## Abstract

Endocrine disrupting chemicals (EDCs) are exogenous substances capable of altering the human hormone system and causing various diseases such as infertility and cancer as a result. In this work, a method for determining twenty-three different EDCs including parabens, alkylphenols, phenylphenols, organophosphorus pesticides, bisphenol A and triclosan in dairy products was developed. Samples are conditioned by addition of acetonitrile containing 1% formic acid, centrifugation and clean-up of the extract by continuous solid-phase extraction. EDCs in the extract are derivatised by heating in a microwave oven and quantified by gas chromatography–mass spectrometry. The proposed method features good limits of detection (6–40 ng/kg) and precision (relative standard deviation < 7.6%); also, it is scarcely subject to matrix effects (1–20%). EDC recoveries from spiked samples ranged from 80 to 108%. The method was used to analyse a total of 33 samples of dairy products including cow, sheep and goat milk, yoghourt, milkshakes, cheese, cream, butter and custard. Bisphenol A was the individual contaminant detected in the greatest number of samples, at concentrations from 180 to 4800 ng/kg. 2-Phenylphenol and ethylparaben were found in more than one-half, at concentrations over the range 130–3500 and 89–4300 ng/kg, respectively. In contrast, alkylphenols, organophosphorus pesticides and triclosan were detected in none.

## 1. Introduction

Compounds with hormonal activity such as endocrine disrupting chemicals (EDCs) have been in the spotlight of food safety research for a while now. EDCs are exogenous substances that interfere with hormone synthesis, release, transmission, binding, excretion, action or clearance in living organisms, where hormones play roles such as maintaining balance, promoting reproduction and development and adjusting various bodily behaviours [1]. The xenoestrogens bisphenol A (BPA), nonylphenol (NP) and 4-*tert*-octylphenol (4-t-OP), which are industrially produced in high volumes [2], are often present in food packaging plastics and other materials from which they can migrate to food and water [1]. Organophosphorus pesticides, another group of EDCs, are monitored on the grounds of their adverse effects on human health [3]. Finally, parabens are widely used as antimicrobial preservatives against moulds and yeasts, as well as in cosmetics and pharmaceuticals and food and beverage processing [4].

Human exposure to endocrine disruptors can have serious health consequences. Thus, EDCs impair physiological reactions of the reproductive system and cause a number of abnormalities including infertility, polycystic ovary syndrome and menstrual irregularities [5]. BPA and parabens have frequently been associated to an increased incidence of breast and prostate cancer [6]. Also, there is evidence that BPA can induce Parkinson’s disease [7].

Dairy products can be contaminated indirectly with EDCs through animal feed, soil, water and air and also during storage and processing, by effect of the contaminants being fat-soluble and hence easily accumulating in human tissues or even being passed onto the offspring [8]. Endocrine disruptors have been encountered in various types of milk and dairy products. Thus, Bemrah et al. (2014) found bisphenol A at concentrations from 45 to 6100 ng/kg [9] in French milk, butter and ultra-fresh dairy products and Al-Julaifi et al. (2015) found organophosphate pesticides at levels from 21,000 to 73,000 ng/kg in Egyptian cheese samples [10].

The presence of ECDs in foods such as dairy products can be hazardous to human health. Regulation (EU) 2019/533 requires the European Union Member States to analyse combinations of pesticides in products including milk over the period 2020–2022 [11]. Also, the European Commission and the Codex Alimentarius have established maximum residue limits (MRLs) of 10–50 µg/kg for pesticides in food and animal products [12,13], and Commission Regulation (EU) 2018/213 has authorised the use of BPA at concentrations below a specific migration limit (SML) of 0.05 mg/kg in food [14]. In June 2020, butylparaben was added to the EU candidate list of substances of very high concern (SVHCs), which currently comprises 209 compounds with potentially severe effects on living organisms and/or the environment [15]. Based on the foregoing, a clear need exists to develop a sensitive, selective, fast method for extracting and detecting EDCs.

Endocrine disruptors in foods with a fatty matrix have so far been determined with chromatographic techniques (especially gas or high performance liquid chromatography in combination with mass spectrometry) [1,16]. Gas chromatography is in fact an effective choice for quantifying EDCs with good separation efficiency and a high throughput [17]. This technique is most often used in combination with single quadrupole (CG–MS) or triple quadrupole mass spectrometry (CG–MS/MS) to detect EDCs [18], the analytes usually being derivatised to improve sensitivity, avoid false positives and protect the chromatographic column [9,19]. Liquid chromatography coupled with a triple quadrupole mass spectrometer (HPLC–MS/MS) is the second choice for determining EDC residues in fatty matrices on account of its high sensitivity and selectivity [20,21]. However, food matrices containing lipids and fats require convenient, flexible methods for increased accuracy. The high complexity of milk and dairy products make extracting EDCs from them especially difficult owing to their solubility in the lipid fraction, and usually requires extract clean-up and analyte concentration [1]. EDCs in food samples are usually extracted by solid-phase extraction (SPE) with an appropriate sorbent [16,22,23,24]. Alternative techniques for this purpose include liquid–liquid extraction [21], dispersive liquid–liquid microextraction [24], QuEChERS [10,18,20], matrix solid-phase dispersion extraction [25] and dispersive micro-solid phase extraction [26].

The primary aim of this work is to develop and validate an analytical method for quantifying twenty-three different EDCs including seven parabens (metylparaben, ethylparaben, propylparaben, isopropylparaben, butylparaben, isobutylparaben and benzylparaben), ten organophosphorus pesticides (dichlovos, dimethoate, diazinon, bromophos methyl, chloropyrifos, fenthion, fenthion sulphoxide, parathion methyl, malathion and methidathion), two alkyphenols (nonylphenol and 4-*tert*-ocylphenol), two phenylphenols (2-phenylphenol and 4-phenylphenol), BPA and triclosan (TCS) in milk and dairy products. The fatty matrices of the products were removed by using an environmentally friendly procedure that uses minimal volumes of organic solvents in combination with a closed system to prevent contamination. The proposed analytical method involves the prior removal of the sample matrix by adding acetonitrile containing 1% formic acid to precipitate fats and proteins, mainly, centrifugation and clean-up of the extract by continuous solid-phase extraction. For improved results, the analytes are subjected to microwave-assisted derivatisation prior to determination by GC–MS. The method was assessed for performance in terms of recovery, precision, linearity and limits of detection and successfully used to quantify the target EDCs in real samples of milk and dairy products from Spain. The objective of the optimisation studies is to obtain a method that significantly simplifies the cleaning stage and that is also more sensitive, selective, precise and accurate than existing methodologies for the determination of target analytes in the matrices indicated above.

## 2. Materials and Methods

### 2.1. Reagents

Analytical standards for the parabens (metylparaben, ethylparaben, propylparaben, isopropylparaben, butylparaben, isobutylparaben and benzylparaben), organophosphorus pesticides (dichlorvos, diazinon, dimethoate, bromophos methyl, chloropyrifos, fenthion, fenthion sulphoxide, parathion methyl, malathion and methidathion), alkyphenols (nonylphenol and 4-*tert-*ocylphenol), phenylphenols (2-phenylphenol and 4-phenylphenol), BPA and TCS, in addition to triphenylphosphate (internal standard, IS), were all purchased from Sigma–Aldrich (St. Louis, MO, USA) in the highest available purity. Other reagents such as trimethylchlorosilane (TMCS) and *N*,*O*-*bis*(trimethylsilyl)trifluoroacetamide (BSTFA) were obtained from Fluka (St. Louis, MO, USA).

Methanol (MeOH), ethanol, acetonitrile (ACN), *n*-hexane, acetone, ethyl acetate, dichloromethane, 2-propanol and *N*-*N*-dimethylformamide (DMF) (HPLC-grade) were obtained from Merck (Darmstadt, Germany). The polymeric sorbent LiChrolut EN (*N*-vinyl pyrrolidone/divinylbenzene copolymer, particle size 40–120 μm) was also supplied by Merck. Formic acid (FA, 98% pure), hydrochloric acid (HCl, 37% pure) and sodium hydroxide (NaOH, 85% pure) were purchased from Fluka (St. Louis, MO, USA). A Millex LG filter unit (hydrophilic, PTFE, pore size 0.20 µm, diameter 25 mm, filtration area 3.9 cm^2^) was supplied by Millipore (Bedford, MA, USA). Finally, ultra-pure water was from a Milli-Q system, also from Millipore.

All stock solutions were prepared individually by dissolving a 5 g/L concentration of each compound in methanol and stored in glass-stopped bottles at 4 °C in the dark. The stocks were used to prepare working-strength solutions by appropriate dilution with purified or uncontaminated samples on a daily basis.

### 2.2. Equipment

Gas chromatography–mass spectrometry analyses were performed on a Focus instrument from Thermo Electron (Madrid, Spain) coupled to a single-quadrupole DSQ II mass spectrometer operating in the electron ionisation (EI) mode. Samples were injected via an AL/AS 3000 autosampler from Thermo Scientific. The chromatograph was furnished with an HP-5MS capillary column (30 m long, 0.25 mm i.d., 0.25 µm film thickness) and He at 1.0 mL/min was used as the carrier gas.

The GC–MS conditions used are as follows: after 1 min at 70 °C, the oven temperature was raised to 150 °C at 14 °C/min, then to 215 °C at 6 °C/min and, finally, to 285 °C at 10 °C/min. The ion source and quadrupole temperatures were both 200 °C, and the mass spectrometer was used at 70 eV in the electron impact mode (EI). The temperature of the transfer line was set at 280 °C and solvent delay at 6 min. Injection was done in the split/splitless mode, using a sample volume of 1 μL in each run. Detection analyses were done in the selected ion monitoring (SIM) mode, using at least three characteristic ions for each analyte (Table 1).

Samples were centrifuged on Centrofriger BL-II apparatus from JP Selecta (Barcelona, Spain) and SPE done with a Minipuls-3 peristaltic pump from Gilson (Viliers le Bel, France). The SPE system comprised two injection valves, polyvinyl chloride tubing and a laboratory-made PTFE column packed with 80 mg of LiChrolut EN. The sorbent material was cleaned and conditioned with 1 mL each of ACN, *n*-hexane, MeOH and ultra-pure water between runs. Under these conditions, the column remained usable with no appreciable loss of performance for at least 1–2 months.

### 2.3. Sample Collection and Preparation

**Figure 1:**
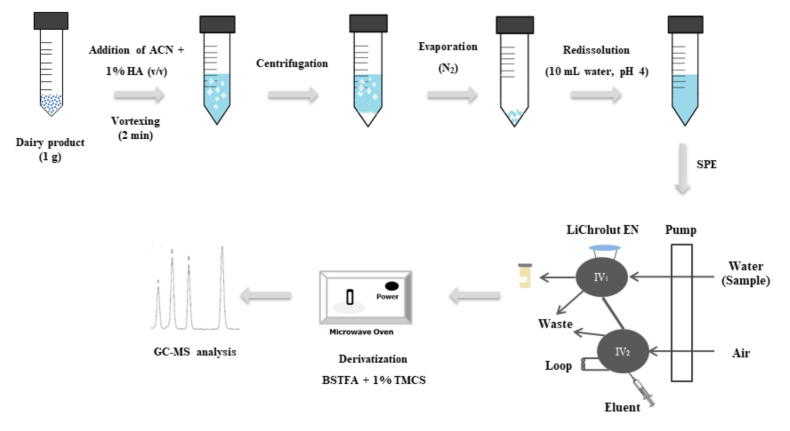
Procedure for determining endocrine disrupting chemicals in dairy products. BSTFA *N*,*O*-*bis*(trimethylsilyl)trifluoroacetamide; GC–MS gas chromatography–mass spectrometry; IV injection valve; SPE solid-phase extraction; TMCS trimethylchlorosilane.

Cow, sheep and goat milk, yoghourt, cream, butter, milkshakes, custard and cheese samples were purchased at different supermarkets in Spain and stored refrigerated at 4 °C in the dark until analysis. All samples were conditioned as described below for analysis. First, they were homogenised in a 50 mL propylene Falcon tube at room temperature. Then, 1 g of sample was placed in another tube, supplied with 8 mL of ACN containing 1% FA (*v*/*v*) and vortexed for 2 min. Next, the mixture was centrifuged at 5000 rpm (2150× *g*) at 4 °C for 10 min, and the supernatant was transferred to a 15 mL glass test tube, passed through a 0.20 µm Millex-LG filter and carefully evaporated to near-dryness (∼200 µL) under a nitrogen stream. The residue thus obtained was redissolved with 10 mL of ultra-pure water and the pH adjusted to 4 with 4 M HCl or 1 M NaOH as required. The reconstituted extract was then purified and preconcentrated on a semi-automated SPE system including a laboratory-made column packed with 80 mg of LiChrolut that was placed in the loop of injection valve IV_1_ (Figure 1). The whole volume was passed through the column at 5.0 mL/min and the analytes were retained in the sorbent while the matrix sample was sent to waste. Then, the sorbent was dried with an air stream circulated at 5 mL/min for 3 min in both ways (forward and reverse) and a volume of 450 µL of eluent containing IS (500 µg/L triphenylphosphate in ACN) was injected into the SPE system via the loop of IV_2_. The eluate was collected in an air-tight 0.5 mL conical glass insert and evaporated to ~25 µL under a stream of ultra-pure nitrogen. Next, 70 µL of BSTFA + 1% TMCS mixture was added and the vial tightly sealed. Finally, the analytes were derivatised in a household microwave oven at 350 W for 3 min [27], and 1 µL of the resulting solution was injected into the GC–MS equipment for analysis.

## 3. Results and Discussion

### 3.1. Optimisation of Sample Treatment

**Figure 2:**
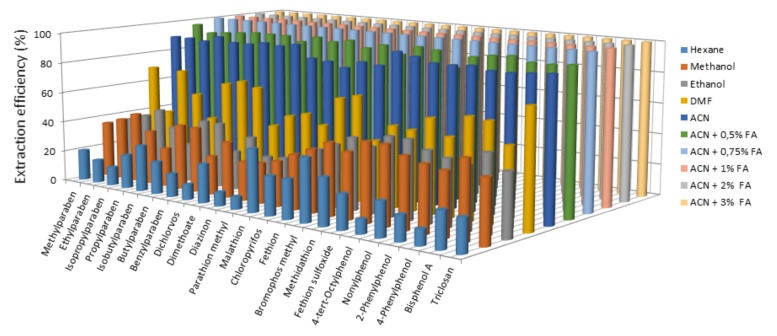
Influence of the solvent on the extraction of endocrine disrupting chemicals from dairy products.

Removing contaminants from highly complex matrices such as those of dairy products requires using a highly efficient extraction procedure. Because dairy products contain high proportions of proteins (0.5–24%) and lipids (0.3–82%) [28], their removal requires using efficient solvents to dissolve and precipitate these two fractions. With EDCs, the choices include liquid–liquid extraction, solid-phase extraction, solid-phase micro-extraction, dispersive liquid–liquid micro-extraction, microwave-assisted extraction and QuEChERS [1]. In this work, we used a procedure involving liquid–liquid extraction followed by extract clean-up in a continuous solid-phase extraction system (Figure 1). The liquid–liquid extraction step was optimised in terms of organic solvent, which was chosen from *n*-hexane, MeOH, EtOH, DMF and ACN, the last both individually and in mixtures with FA [4]. For this purpose, a 500 ng/kg concentration of each EDC was added to an amount of 1 g of milk or dairy product (yoghourt, cream, butter, milkshakes, custard and cheese), mixed with 8 mL of solvent, vortexed for 2 min and centrifuged at 5000 rpm (2150× *g*) at 4 °C for 10 min. After proteins and lipids were removed, the extracts were evaporated to dryness under a nitrogen stream, the residues being reconstituted with ultra-pure water for SPE and derivatisation of analytes as described in Section 2.3. The most efficient solvent as regards extraction was ACN containing FA (Figure 2). The optimum proportion of FA to be used was examined over the range 0.5–3% (*v*/*v*). As can be seen in Figure 2, the extraction efficiency increased with increasing proportion of FA up to 0.75% (*v*/*v*). Therefore, acetonitrile containing 1% FA was selected as extractant for further testing.

The optimum volume for analyte extraction was established by spiking some samples with a 500 ng/kg concentration of each EDC and extracting them with variable volumes of ACN/1% FA mixture from 1 to 15 mL. The extraction efficiency increased with increasing extractant volume up to 7 mL, above which it levelled off. A volume of 8 mL of ACN containing 1% FA was thus selected. The influence of centrifugation-related variables (i.e., time, speed and temperature) was examined in the ranges 1–25 min, 1000–5000 rpm (86–2150× *g*) and 0–15 °C, respectively, and the optimum values were found to be 10 min, 5000 rpm (2150× *g*) and 4 °C, respectively.

As noted earlier, clean-up of the extracts before insertion into the GC–MS equipment was needed to prevent residual sample matrix from impairing the performance of the chromatographic column—and hence identification and quantification of the analytes by MS. Solid-phase extraction is the most widely used clean-up technique to minimise matrix effects on food extracts, avoid interferences and prevent instrument failure. In previous work, our group assessed the efficiency of different sorbent materials including LiChrolut EN, Oasis-HLB, RP-C18 and Amberlites XAD-2 and XAD-4, in the SPE of EDCs, using an amount of 80 mg of each sorbent in laboratory-made columns [27]. We found LiChrolut EN to be the best column-packing material for the intended purpose. The influence of pH on SPE performance was examined over the range 1–10 by using HCl and NaOH solutions of appropriate concentrations. Extraction of analytes was maximal at pH 3–5, so pH 4 was selected for further testing. Eluting the analytes retained in the sorbent column with MeOH, acetone, ACN, ethyl acetate, *n*-hexane, dichloromethane and 2-propanol revealed that ACN was the most efficient solvent as it eluted all EDCs almost quantitatively. The influence of the eluent volume was examined over the range 100–650 μL by changing the loop of IV2 in SPE system (Figure 1) as required. Volumes greater than 400 μL resulted in quantitative elution, so 450 μL was chosen for further testing. Finally, the extract was evaporated to ~25 μL and the analytes were derivatised with 70 µL of BSTFA + 1% TMCS as described in Section 2.3.

### 3.2. Matrix Effects

Matrix effects (ME) should always be considered in developing an analytical method for complex samples. In this work, they were assessed by comparing the slopes of matrix-matched calibration curves with those of external standard calibration curves through the following equation [29]:

ME = [(slope of matrix-matched curve/slope of in-solvent curve) − 1] × 100(1)

The sample matrix can diminish analytical signals and result in negative ME values; however, it can also boost signals. Gilbert-López et al. (2010) classified matrix effects as negligible, mild, moderate and strong according to whether ME is 0–10%, 10–20%, 20–50% and >50%, respectively [30]. As can be seen in Table 2, 52.8% of our ME values fell in the range 0–10% and the remainder in the 11–20% range. Therefore, the clean-up procedure used was specific enough to avoid substantial matrix effects.

### 3.3. Analytical Performance

The proposed SPE–GC–MS method was validated in terms of regression coefficient, linear range, analyte detectability and relative standard deviation for the body of EDCs. Uncontaminated samples were used as blanks to assess selectivity, and spiked samples to identify the analyte peaks and estimate precision, linearity and accuracy.

**Figure 3:**
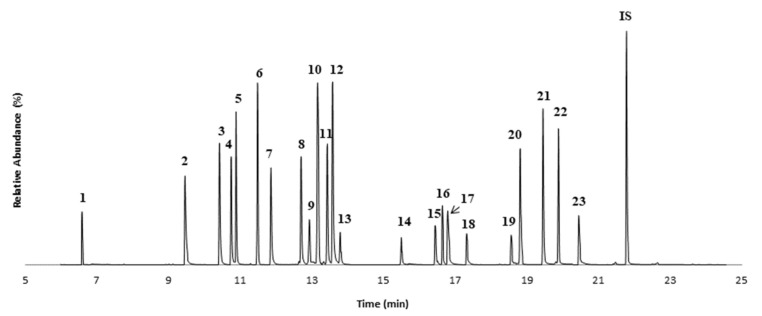
Typical chromatogram in the SIM mode for 1 g of skimmed cow milk spiked with a 500 ng/kg concentration of each EDC. 1, dichlorvos; 2, methylparaben; 3, ethylparaben; 4, isopropylparaben; 5, 2-phenylphenol; 6, 4-*tert*-octylphenol; 7, propylparaben; 8, isobutylparaben; 9, dimethoate; 10, nonylphenol; 11, butylparaben; 12, 4-phenylphenol; 13, diazinon; 14, parathion methyl; 15, malathion; 16, chloropyrifos; 17, fenthion; 18, bromophos methyl; 19, methidathion; 20, triclosan; 21, benzylparaben; 22, bisphenol A; 23, fenthion sulphoxide; IS, internal standard.

Linearity was assessed by adding appropriate volumes of working-strength solutions at concentrations from 20 to 10,000 ng/kg to uncontaminated skimmed cow milk and butter samples, and processing them as described in Section 2.3. An acceptable correlation coefficient (0.993–0.999) was thus obtained for all analytes (Table 1). By way of example, Figure 3 shows a typical chromatogram for a skimmed cow milk sample used to construct the calibration curves.

The sensitivity of the method was assessed in terms of limits of detection (LODs), which were calculated as three times the standard deviation of background noise divided by the slope of each calibration graph. LODs ranged from 6 to 40 ng/kg. Limits of quantification (LOQs) were taken to be 3.3 times the previously calculated LODs (Table 1).

The precision of the method, as relative standard deviation (RSD), was determined by analysing 11 samples of milk and dairy products spiked with EDCs at three different concentrations (200, 500 and 1500 ng/kg). Analyses were performed on the same day (within-day precision) or on seven different days (between-day precision). As can be seen in Table 2, the seven types of samples used provided good results: within-day precision was 2.1–6.9% and between-day precision 2.8–7.6%.

The accuracy of the proposed method was assessed by in-house validation. For this purpose, samples of the dairy products were spiked with 200, 500 and 1500 ng/kg concentrations of each analyte in triplicate. Since most samples contained some EDC, analyte recoveries were calculated by subtracting the previously determined EDC concentrations from their total contents. All EDCs were thus accurately identified; also, average recoveries were acceptable (80–108%) (Table 3), which were consistent with the results of the matrix effect determinations (Section 3.2).

### 3.4. Analysis of Real Samples

The proposed method was validated by simultaneously determining all EDCs in whole, semi-skimmed and skimmed cow, sheep and goat milk and also in other dairy products (yoghourt, butter, cheese, custard, cream and milkshakes). Each sample was analysed in triplicate and followed by a blank. In each run, 1 g of sample was subjected to the procedure described in Section 2.3. The results are shown in Table 4, which include the fat and protein contents of the samples. Of the 32 samples studied, only 5 (skimmed cow milk no. 4, butter no. 3 and the three custard samples) contained none of the analytes—at least at levels above the detection threshold of the proposed method.

As can be seen from Table 4, BPA was the analyte detected in the greatest number of samples, at concentrations from 180 to 4800 ng/kg—and at especially increased levels in cow milk. These BPA concentrations are lower than those obtained by Bemrah et al. (2014) in dairy products (45–6100 ng/kg) [9] and Xiong et al. (2018) in milk (13,700 ng/kg) [31] (Table 5); however, they clearly exceed the BPA concentration in milk as reported by Shao et al. (2007): 490 ng/kg [25]. As per Regulation (EU) 2018/213, BPA concentrations must not exceed the SML for this compound: 50,000 ng/kg [14].

2-Phenylphenol was detected in 53% of the samples, at levels from 130 to 3500 ng/kg that were especially high in two butter samples. To our knowledge, this is the first report of the presence of 2-phenylphenol, a compound that was previously found in breast milk samples (Table 5) [4,23], in dairy products. This compound is a widely used antimicrobial agent and agricultural fungicide for the surface treatment of citrus fruit as food additive E231 [32]; also, it is frequently used as a surface disinfectant for granaries, food plants, households, farms, laundries and social institutions, among others. By contrast, 4-phenylphenol was present in only three types of samples (viz., fresh cow milk, whole sheep milk and fresh goat milk), at concentrations from 130 to 230 ng/kg.

Parabens possess anti-microbial and anti-fungal properties that have long been used to avoid microbial contamination and prevent degradation of active ingredients [5]. Ethylparaben, the most ubiquitous of all in the samples, was present in more than one-half, at concentrations from 89 to 4300 ng/kg (Table 4). Methylparaben was detected in only one type of sample (cheese), at 170 ng/kg, whereas butylparaben was present in fresh goat milk and in cream (240–620 ng/kg). The previous parabens, in addition to propylparaben, were found by Dualde et al. (2019) at concentrations from 110 to 7000 ng/L in human milk [33]. Ye et al. (2008) detected methylparaben and propylparaben at similar levels (320–3040 ng/L) in human milk [4], and our group previously found six types of parabens at levels from 150 to 8100 ng/L in human milk [23].

Other EDCs such as organophosphorus pesticides, alkylphenols and TCS were found in none of the samples even though, as can be seen in Table 4, they were previously detected in dairy products by other authors [10,23,25,34].

## 4. Conclusions

A total of twenty-three ECDs including parabens, organophosphorus pesticides, alkylphenols, phenylphenols, BPA and TCS were determined in dairy products by using an environmentally friendly procedure involving SPE for clean-up of samples and preconcentration of analytes, and GC–MS for determination. Removal of the analytes from the sample matrix was carefully optimised. The precision, recovery, linearity and limits of detection of the proposed method make it useful to determine the analytes in milk and dairy products. In fact, as can be seen from Table 5, the method is very sensitive; thus, its limits of detection (6–40 ng/kg) are on a par with those of Shao et al. (2007) [25] and Dualde et al. (2019) [33], who used HPLC–MS/MS to determine bisphenols, parabens and alkylphenols in milk samples (Table 5).

The method was successfully used to analyse various types of dairy products including semi-skimmed, skimmed milk, whole and fresh milk, milkshakes, custard, cheese, cream and butter. Although 44% of samples were found to contain BPA, its concentrations never exceeded the limit set by Regulation (EU) 2018/213 [14]. The most likely source of BPA was contact of the foods with their packaging material. Worth special note is the presence of ethylparaben and 2-phenylphenol in most samples, at concentrations over the range 89–4300 ng/kg and 130–3500 ng/kg, respectively. In contrast, organophosphorus pesticides, TCS and alkyphenols were detected in none. Overall, our results suggest that ECDs are present in fairly small amounts, if any, in dairy products, and also that their presence results from mild to moderate contamination of feed given to milk-producing animals, contact of the foods with their packaging or disinfection of food processing equipment.

## Figures and Tables

**Table 1:** Analytical figures of the determination of EDCs in milk and dairy products by the proposed SPE–GC–MS method ^a^.

						*m*/*z*		
EDCs	Compounds	LOD (ng/kg)	r	Linear Range (ng/kg)	t_R_	[M]^+^	[M − 15]^+^	Additional Ions
**Parabens**	Methylparaben	12	0.994	40–10,000	9.47	224	**209**	135, 149, 177, 193
	Ethylparaben	9	0.994	30–10,000	10.41	238	**223**	135, 151, 193
	Isopropylparaben	10	0.995	33–10,000	10.75	252	237	151, **193**, 195, 210
	Propylparaben	11	0.996	36–10,000	11.87	252	237	**193**, 195, 210
	Isobutylparaben	10	0.997	34–10,000	12.70	266	251	151, **193**, 195, 210
	Butylparaben	9	0.995	30–10,000	13.41	266	251	193, 195, **210**
	Benzylparaben	7	0.997	23–10,000	19.50	300	285	91, **193**, 255
**Organophosphorus** **pesticides**	Dichlorvos	21	0.993	70–10,000	6.61	220	–	**109**, 145, 185
	Dimethoate	23	0.996	76–10,000	12.90	229	–	187, 93, **125**, 143
	Diazinon	32	0.996	105–10,000	13.78	304	–	137, **179**, 199
	Parathion methyl	40	0.999	135–10,000	15.54	**263**	–	109, 200
	Malathion	28	0.994	92–10,000	16.46	332	–	93, 125, 158, **173**
	Chloropyrifos	18	0.995	60–10,000	16.65	349	–	199, 258, 286, **314**
	Fenthion	20	0.998	65–10,000	16.78	**278**	–	109, 125, 169
	Bromophos methyl	35	0.995	115–10,000	17.33	366	–	109, 125, 213, **331**
	Methidathion	36	0.996	120–10,000	18.57	302	–	85, 125, **145**
	Fenthion sulfoxide	22	0.998	73–10,000	20.47	294	–	125, 169, **279**
**Alkyphenols**	4-*tert*-Octylphenol	6	0.995	20–10,000	11.50	278	263	151, 191, **207**
	Nonylphenol	6	0.994	20–10,000	13.15	292	277	179, **207**, 221, 263
**Phenylphenols**	2-Phenylphenol	7	0.996	23–10,000	10.89	242	**227**	105, 152, 211
	4-Phenylphenol	6	0.994	20–10,000	13.65	**242**	227	113, 152, 207, 211
**Others**	Bisphenol A	8	0.998	26–10,000	19.82	372	**357**	207, 285
	Triclosan	9	0.996	30–10,000	18.82	362	**347**	200, 310

^a^ LOD: limit of detection; r: correlation coefficient; t_R_: retention time; *m*/*z*: mass/charge ratio; [M]^+^: ionised mass, [M − 15]^+^: loss of a CH3 radical from the Si(CH3)_3_ group; –: analyte non-derivatised; the peaks used for quantification are boldfaced.

**Table 2:** Performance of the sample treatment procedure in terms of precision as relative standard deviation (RSD, %) and matrix effects.

Compounds	RSD (%) ^a^														Matrix Effect (%) ^b^						
	Milk		Yoghourt		Custard		Butter		Cream		Shakes		Cheese		Milk	Yoghourt	Custard	Buttter	Cream	Shakes	Cheese
	WD	BD	WD	BD	WD	BD	WD	BD	WD	BD	WD	BD	WD	BD							
Methylparaben	4.8	5.6	5.0	5.9	5.2	6.2	4.8	5.7	5.5	6.9	6.0	6.5	5.4	6.4	0.94 (−6%)	0.85 (−15%)	0.88 (−12%)	1.07 (+7%)	0.83 (−17%)	1.08 (+8%)	1.19 (+19%)
Ethylparaben	4.5	5.3	5.6	6.6	5.8	6.9	5.7	6.7	3.8	7.5	4.6	6.5	3.9	4.6	1.01 (+1%)	1.08 (+8%)	0.83 (−17%)	0.90 (−10%)	0.86 (−14%)	0.98 (−2%)	1.06 (+6%)
Isopropylparaben	5.7	6.7	5.8	6.8	6.0	7.1	3.3	3.9	5.0	5.8	6.0	6.9	3.7	4.4	0.91 (−9%)	1.11 (+11%)	0.98 (−2%)	0.93 (−7%)	0.94 (−6%)	1.15 (+15%)	0.91 (−9%)
Propylparaben	5.5	6.5	2.7	3.2	2.8	3.5	5.5	6.5	3.1	6.1	2.9	3.7	3.9	4.6	1.08 (+8%)	0.87 (−13%)	0.88 (−12%)	0.86 (−14%)	0.91 (−9%)	0.91 (−9%)	1.14 (+14%)
Isobutylparaben	3.1	3.7	3.4	4,0	3.6	4.3	5.1	6.0	4.6	7.6	3.4	3.9	5.3	6.2	0.83 (−17%)	0.83 (−17%)	1.09 (+9%)	0.89 (−11%)	1.01 (+8%)	0.98 (−2%)	0.80 (−20%)
Butylparaben	5.8	6.8	2.2	3.6	2.6	4.7	5.4	6.4	3.6	4.6	2.9	3.6	4.8	5.7	0.86 (−14%)	0.94 (−6%)	1.06 (+6%)	0.84 (−16%)	0.86 (−14%)	1.11 (+11%)	0.87 (−13%)
Benzylparaben	5.7	6.7	2.4	2.8	2.8	2.9	5.4	6.3	3.8	4.2	3.4	4.8	5.8	6.8	0.84 (−16%)	0.83 (−17%)	0.89 (−11%)	1.18 (+18%)	0.87 (−13%)	1.03 (+3%)	0.86 (−14%)
Dichlorvos	5.8	6.8	5.4	6.3	5.6	6.4	4.3	5.0	4.6	7.0	5.5	6.3	5.0	5.9	1.04 (+4%)	0.85 (−15%)	0.83 (−17%)	0.92 (−8%)	1.15 (+15%)	0.89 (−11%)	1.13 (+13%)
Dimethoate	4.9	5.8	4.5	5.3	4.7	5.4	4.8	5.6	4.8	5.5	6.0	7.3	5.8	6.8	0.96 (−4%)	1.12 (+12%)	1.10 (+10%)	0.88 (−12%)	0.87 (−17%)	1.01 (+1%)	0.89 (−11%)
Diazinon	6.0	7.1	6.0	7.0	6.6	7.1	5.4	6.4	5.9	7.5	6.3	6.9	5.9	6.9	0.87 (−13%)	0.98 (−2%)	0.86 (−14%)	1.17 (+17%)	0.98 (−2%)	0.85 (−15%)	1.13 (+13%)
Parathion methyl	4.8	5.6	2.7	3.2	3.3	3.6	5.9	6.9	4.3	6.0	3.7	5.3	6.0	7.0	0.98 (−2%)	0.85 (−15%)	0.83 (−17%)	1.14 (+14%)	1.05 (+5%)	0.96 (−4%)	1.12 (+12%)
Malathion	5.9	6.9	3.5	4.1	4.1	4.5	5.4	6.4	5.1	5.9	5.5	6.5	4.3	5.1	1.15 (+15%)	1.05 (+5%)	0.87 (−13%)	0.96 (−4%)	0.79 (−19%)	0.91 (−9%)	1.03 (+3%)
Chloropyrifos	4.4	5.2	6.3	7.4	6.9	7.5	5.9	6.9	3.9	6.3	6.4	7.0	6.4	7.5	0.81 (−19%)	1.03 (+3%)	0.92 (−8%)	1.15 (+15%)	1.10 (+10%)	0.88 (−12%)	1.15 (+15%)
Fenthion	6.0	7.0	5.4	6.3	6.0	6.9	5.6	6.6	5.0	6.3	3.4	6.5	6.1	7.2	1.08 (+8%)	1.11 (+11%)	0.91 (−9%)	1.12 (+12%)	0.90 (−10%)	0.88 (−12%)	1.06 (+6%)
Bromophos methyl	5.2	6.1	4.3	5.0	4.9	5.1	2.8	3.8	2.9	5.6	3.3	5.0	5.4	6.4	1.13 (+13%)	0.85 (−15%)	0.85 (−15%)	0.96 (−4%)	1.08 (+8%)	0.94 (−6%)	1.09 (+9%)
Methidathion	5.2	6.1	4.1	4.8	4.7	4.9	5.8	6.8	4.7	6.2	2.1	5.8	5.3	6.2	1.12 (+12%)	0.95 (−5%)	0.91 (−9%)	1.10 (+10%)	1.01 (+1%)	0.85 (−15%)	1.01 (+1%)
Fenthion sulphoxide	5.0	5.9	4.3	5.0	4.7	5.1	5.0	5.9	3.7	5.0	4.5	5.1	5.9	6.9	1.01 (+1%)	0.93 (−7%)	1.19 (+19%)	0.87 (−13%)	1.08 (+8%)	0.85 (−15%)	1.19 (+19%)
4-tert-Octylphenol	6.0	7.1	2.4	2.8	2.6	3.1	5.9	6.9	2.6	2.9	4.4	5.8	5.2	6.8	0.85 (−15%)	1.17 (+17%)	1.06 (+6%)	0.86 (−14%)	1.15 (+15%)	1.01 (+1%)	0.92 (−8%)
Nonylphenol	5.9	6.9	5.8	6.8	6.0	6.9	3.4	4.0	5.7	7.0	6.8	7.3	6.0	7.1	1.04 (+4%)	1.13 (+13%)	1.01 (+1%)	1.04 (+4%)	0.87 (−13%)	0.86 (−14%)	0.85 (−15%)
2-Phenylphenol	6.0	7.0	6.0	7.0	6.2	7.3	4.6	5.4	3.2	6.4	6.2	7.1	4.2	7.1	0.99 (−1%)	1.08 (+8%)	0.92 (−8%)	1.12 (+12%)	0.87 (−13%)	1.01 (+1%)	0.88 (−12%)
4-Phenylphenol	4.1	4.8	2.4	2.8	3.2	2.9	6.0	7.0	3.3	4.7	2.8	4.8	5.7	6.7	0.79 (−19%)	1.14 (+14%)	0.86 (−14%)	0.89 (−11%)	1.08 (+8%)	0.83 (−17%)	0.91 (−9%)
Bisphenol A	3.9	4.6	3.3	4.5	2.7	3.6	5.7	6.7	2.7	4.9	2.3	4.5	5.2	6.1	1.14 (+14%)	0.99 (−1%)	0.90 (−10%)	0.94 (−6%)	0.78 (−16%)	0.85 (−15%)	1.02 (+2%)
Triclosan	2.6	3.1	3.0	3.5	3.4	3.6	4.3	5.1	3.4	4.5	3.5	5.5	4.9	5.8	0.94 (−6%)	1.02 (+2%)	0.97 (−3%)	0.85 (−15%)	1.18 (+18%)	0.91 (−9%)	1.16 (+16%)

^a^ WD: within-day; BD: between-day. ^b^ Matrix effects are expressed as the ratio between the calibration curve slope in matrix and calibration curve slope in solvent. The result of the following operation is included in parentheses: [(calibration curve slope in matrix/calibration curve slope in solvent) − 1] × 100.

**Table 3:** Average recoveries (% ± SD, *n* = 3) of endocrine disrupting chemicals spiked to milk and dairy products.

Compounds		Milk		Yoghourt		Custard		Butter		Cream		Shakes		Cheese	
		200 ng/kg	500 ng/kg	200 ng/kg	500 ng/kg	200 ng/kg	500 ng/kg	200 ng/kg	500 ng/kg	200 ng/kg	500 ng/kg	200 ng/kg	500 ng/kg	200 ng/kg	500 ng/kg
**Parabens**	Methylparaben	106 ± 6	99 ± 5	86 ± 4	99 ± 8	98 ± 6	98 ± 7	86 ± 5	99 ± 6	104 ± 5	100 ± 9	96 ± 5	105 ± 4	101 ± 5	99 ± 5
	Ethylparaben	89 ± 7	91 ± 5	85 ± 4	102 ± 5	86 ± 4	100 ± 5	84 ± 5	101 ± 5	105 ± 6	101 ± 7	104 ± 7	100 ± 5	89 ± 6	99 ± 5
	Isopropylparaben	104 ± 6	84 ± 5	103 ± 6	101 ± 6	80 ± 4	99 ± 5	96 ± 5	94 ± 5	94 ± 7	94 ± 5	106 ± 6	99 ± 6	104 ± 6	81 ± 4
	Propylparaben	88 ± 5	100 ± 7	85 ± 4	95 ± 5	101 ± 5	95 ± 5	83 ± 5	102 ± 6	89 ± 5	85 ± 4	93 ± 5	103 ± 6	85 ± 5	105 ± 7
	Isobutylparaben	86 ± 7	89 ± 6	88 ± 2	100 ± 3	82 ± 1	102 ± 6	81 ± 4	99 ± 5	102 ± 6	99 ± 6	91 ±5	95 ± 5	87 ± 3	101 ± 6
	Butylparaben	103 ± 5	88 ± 5	105 ± 5	99 ± 5	108 ± 7	98 ± 5	82 ± 4	88 ± 5	105 ± 7	99 ± 5	88 ± 5	89 ± 5	82 ± 5	107 ± 6
	Benzylparaben	85 ± 4	101 ± 5	92 ± 5	98 ± 6	105 ± 6	97 ± 6	88 ± 5	99 ± 5	88 ± 4	105 ± 5	98 ± 7	90 ± 5	102 ± 6	88 ± 4
**Organophosphorus Pesticides**	Dichlorvos	90 ± 5	103 ± 6	92 ± 5	99 ± 7	102 ± 6	99 ± 6	88 ± 5	86 ± 5	106 ± 6	89 ± 5	98 ± 6	96 ± 6	98 ± 7	105 ± 7
	Dimethoate	92 ± 6	91 ± 6	102 ± 8	101 ± 6	101 ± 6	100 ± 6	101 ± 5	98 ± 6	99 ± 6	101 ± 7	101 ± 7	108 ± 7	95 ± 7	99 ± 5
	Diazinon	86 ± 5	106 ± 6	83 ± 4	99 ± 5	86 ± 5	89 ± 5	89 ± 5	106 ± 7	86 ± 5	99 ± 7	89 ± 5	102 ± 6	86 ± 5	100 ± 6
	Parathion methyl	105 ± 6	95 ± 5	106 ± 6	105 ± 6	99 ± 7	100 ± 7	86 ± 4	95 ± 6	96 ± 6	101 ± 6	96 ± 5	95 ± 6	103 ± 6	87 ± 5
	Malathion	101 ± 6	105 ± 5	93 ± 5	83 ± 4	89 ± 4	93 ± 5	83 ± 4	100 ± 7	103 ± 7	86 ± 5	83 ± 5	99 ± 6	82 ± 4	89 ± 5
	Chloropyrifos	95 ± 6	101 ± 6	98 ± 6	108 ± 7	101 ± 7	99 ± 6	104 ± 6	89 ± 5	90 ± 5	89 ± 5	104 ± 6	99 ± 6	97 ± 6	108 ± 7
	Fenthion	107 ± 6	97 ± 6	89 ± 5	99 ± 5	88 ± 4	100 ± 6	101 ± 7	86 ± 4	95 ± 6	98 ± 6	102 ± 7	96 ± 5	106 ± 6	100 ± 6
	Bromophos methyl	88 ± 5	101 ± 6	103 ± 5	102 ± 8	93 ± 6	99 ± 6	99 ± 6	101 ± 6	87 ± 4	106 ± 6	99 ± 6	101 ± 6	90 ± 5	100 ± 6
	Methidathion	85 ± 5	89 ± 5	106 ± 7	100 ± 5	87 ± 4	89 ± 5	101 ± 6	99 ± 7	86 ± 5	89 ± 5	99 ± 7	95 ± 6	100 ± 6	99 ± 7
	Fenthion sulphoxide	99 ± 6	100 ± 6	95 ± 5	99 ± 6	105 ± 6	107 ± 6	89 ± 4	101 ± 6	98 ± 6	90 ± 5	90 ± 5	103 ± 7	98 ± 5	85 ± 4
**Alkyphenols**	4-tert-Octylphenol	84 ± 4	99 ± 5	102 ± 5	100 ± 5	105 ± 7	101 ± 5	101 ± 3	89 ± 4	87 ± 4	89 ± 5	103 ± 6	99 ± 6	108 ± 6	88 ± 4
	Nonylphenol	106 ± 7	97 ± 5	89 ± 5	95 ± 6	102 ± 7	89 ± 5	106 ± 7	87 ± 5	106 ± 6	89 ± 5	104 ± 6	97 ± 6	105 ± 6	101 ± 7
**Phenylphenols**	2-Phenylphenol	97 ± 6	100 ± 6	99 ± 6	98 ± 7	86 ± 4	88 ± 5	100 ± 7	95 ± 6	107 ± 6	101 ± 6	100 ± 6	96 ± 7	95 ± 5	94 ± 5
	4-Phenylphenol	84 ± 4	94 ± 5	104 ± 6	81 ± 4	88 ± 4	102 ± 6	87 ± 4	104 ± 6	99 ± 6	99 ± 6	97 ± 6	100 ± 5	82 ± 4	91 ± 5
**Others**	Bisphenol A	97 ± 6	87 ± 4	99 ± 6	92 ± 5	89 ± 5	106 ± 6	107 ± 6	97 ± 7	99 ± 5	108 ± 6	97 ± 5	107 ± 6	102 ± 6	102 ± 6
	Triclosan	105 ± 6	99 ± 5	96 ± 5	101 ± 6	105 ± 6	100 ± 5	96 ± 5	89 ± 5	91 ± 5	95 ± 5	106 ± 6	89 ± 4	93 ± 4	91 ± 4

**Table 4:** EDC contents (mean ± standard deviation, ng/kg, n = 3) found in various types of dairy products ^a^.

Sample	Compounds	Concentration Found (ng/kg)	Sample	Compounds	Concentration Found (ng/kg)	Sample	Compounds	Concentration Found (ng/kg)
**Skimmed cow’s milk 1** **(0.3%/3.3%)**	2-Phenylphenol Bisphenol A	470 ± 30 3400 ± 200	**Whole goat’s milk** (3.9%/3.4%)	Ethylparaben 2-Phenylphenol Bisphenol A	950 ± 60 130 ± 10 980 ± 60	**Cream 1** (18.1%/2.5%)	Ethylparaben	260 ± 10
**Skimmed cow’s milk 2** **(0.3%/3.2%)**	2-Phenylphenol Bisphenol A	1300 ± 100 3900 ± 200	**Fresh goat´s milk** (3.7%/3.4%)	Ethylparaben Butylparaben 2-Phenylphenol 4-Phenylphenol	320 ± 20 620 ± 40 590 ± 40 230 ± 10	**Cream 2** (18.0%/2.4%)	Ethylparaben Butylparaben	800 ± 50 240 ± 10
**Skimmed cow’s milk 3** **(0.3%/3.1%)**	2-Phenylphenol Bisphenol A	960 ± 60 2300 ± 100				**Cream 3** (18.0%/2.1%)	Butylparaben	330 ± 20
**Skimmed cow’s milk 4** **(0.3%/3.1%)**	nd	-	**Yoghourt cow’s 1** (2.6%/3.9%)	Ethylparaben Bisphenol A	89 ± 5 180 ± 10	**Butter 1** (82.0%/0.7%)	2-Phenylphenol	3500 ± 200
**Semi-skimmed cow’s milk 1** **(1.6%/3.9%)**	2-Phenylphenol Bisphenol A	2000 ± 100 3600 ± 200	**Yoghourt cow’s 2** (3.0%/3.5%)	Bisphenol A	990 ± 60	**Butter 2** (82.0%/0.6%)	2-Phenylphenol	2400 ± 100
**Semi-skimmed cow’s milk 2** **(1.6%/3.5%)**	2-Phenylphenol Bisphenol A	510 ± 30 3800 ± 200	**Yoghourt goat´s** (5.4%/4.3%)	Ethylparaben 2-Phenylphenol Bisphenol A	91 ± 6 520 ± 30 4400 ± 300	**Butter 3** (81.0%/0.5%)	nd	-
**Semi-skimmed cow’s milk 3** **(1.6%/4.9%)**	Bisphenol A	4300 ± 300	**Milkshake 1** (1.0%/1.6%)	Ethylparaben	4100 ± 200	**Custard 1** (2.4 %/2.3%)	nd	-
**Whole cow’s milk 1** **(3.6%/3.1%)**	Ethylparaben 2-Phenylphenol Bisphenol A	230 ± 10 440 ± 30 2100 ± 100	**Milkshake 2** (1.0%/1.6%)	Ethylparaben	4300 ± 300	**Custard 2** (2.9 %/3.4%)	nd	-
**Whole cow’s milk 2** **(3.6%/3.2%)**	Ethylparaben Bisphenol A	120 ± 10 3200 ± 200	**Milkshake 3** (1.5%/3.0%)	Ethylparaben Bisphenol A	1600 ± 100 1100 ± 100	**Custard 3** (3.9 %/3.4%)	nd	-
**Whole cow’s milk 3** **(3.6%/3.1%)**	Ethylparaben 2-Phenylphenol Bisphenol A	160 ± 10 500 ± 30 4600 ± 300	**Cheese 1** (12.1%/10.9%)	Ethylparaben 2-Phenylphenol	760 ± 50 1400 ± 100			-
**Fresh cow’s milk** **(3.6%/3.0%)**	Ethylparaben 2-Phenylphenol 4-Phenylphenol	3100 ± 200 800 ± 50 130 ± 10	**Cheese 2** (13.1%/12.9%)	Metylparaben Ethylparaben 2-Phenylphenol	170 ± 10 1300 ± 100 880 ± 50			
**Whole sheep’s milk** **(6.5%/5.4%)**	Ethylparaben 2-Phenylphenol 4-Phenylphenol Bisphenol A	280 ± 20 1200 ± 100 140 ± 10 2500 ± 200	**Cheese 3** (12.9%/10.1%)	Ethylparaben 2-Phenylphenol	920 ± 60 760 ± 50			

^a^ (%, fat content/protein content); nd: not detected.

**Table 5:** Summary of studies reporting the presence of EDCs in dairy products ^a^.

Analytes	Samples	Location	Sample Pretreatment and Extract Clean-Up	Analytical Technique	Analytical Characteristics	Concentration in Real Samples	References
Parabens, triclosan, BPA, 2-phenylphenol and other phenols	Human milk	USA	SPE	HPLC-MS/MS	LOD: 100–400 ng/L RSD: 3.5–16.3% R: 84–119%	Parabens: 320–3040 ng/L Triclosan: 2810–14,500 ng/L BPA: 450–1620 ng/L 2-Phenylphenol: 120–190 ng/L	[4]
BPA	Milk, butter and ultra-fresh dairy products and other foods	France	SPE, derivatisation (NMNTRA)	GC-MS/MS	LOD: 90–210 ng/kg	45–6100 ng/kg	[9]
Diazinon, methidathion, malathion, chloropyrifos, bromophos methyl and other pesticides	Cheese	Egypt	QuEChERS, DSPE	CG-MS/MS	LOD: 5000–50,000 ng/kg R: 79–130%	21,000–73,000 ng/kg	[10]
Fethion, parathion methyl and other pesticides	Milk, butter, cheese and yogurt	Turkey	QuEChERS	GC-MS	LOD: 260–2990 ng/kg RSD: 0.9–17.9% R: 72–120%	ND	[18]
Parabens, TCS, BPA, nonylphenol, octylphenol and other phenols	Human milk and other biological fluid	Spain	SPE, derivatisation (BSTFA + 1% TMCS)	CG/MS	LOD: 1–9 ng/L RSD: 4.4–7.0% R: 86–104%	Phenols: 550–5600 ng/L BPA: 1400–2900 ng/L Parabens: 15–8100 ng/L	[22]
Dichlorvos, diazinon and other pesticides	Milk and other foods	Iran	SPE/DLLME	GC-MS	LOD: 0.5–1 ng/kg RSD: 0.1–11.8% R: 66–102%	ND	[23]
BPA, nonylphenol and octylphenol	Milk and eggs	China	MSPDE	HPLC-MS/MS	LOD: 50–100 ng/kg R: 82–102%	BPA: 490 ng/kg NP: 4240–17,600 ng/kg OP: 100 ng/kg	[25]
BPA	Milk	Spain	DMSPE	HPLC-UV	LOD: 3050 ng/L RSD: 9.1–16% R: 86–99%	ND	[26]
Bisphenols	Milk	China	QuEChERS	HPLC-FLD	LOD: 1000–3100 ng/kg RSD: 2.6–13.0% R: 76–94%	13,700 ng/kg	[31]
Bisphenols and parabens	Human milk	Spain	QuEChERS	HPLC-MS/MS	LOQ: 100–250 ng/L RSD: 5–16% R: 83–115%	Bisphenols: 130–1660 ng/L Parabens: 110–7000 ng/L	[33]
BPA, nonylphenol, *4-tert-*octylphenol and hormones	Milk	China	SPME	HPLC-DAD	LOD: 91–230 ng/L RSD: <10% R: 76–118%	ND	[34]
Diazinon, methidathion, parathion ethyl, parathion methyl and other pesticides	Raw milk	Italy	LLE, SPE	GC-NPD	LOD: 1000–5000 ng/kg R:60–167%	ND	[35]
Diazinon, chlorpyrifos, dimethoate, fethion sulphoxide, malathion, parathion methyl and other pesticides	Cheese and other foods	China	QuEChERS	HPLC-MS/MS	LOD: 2000 ng/kg RSD: <20% R: 70–120%	ND	[36]
Organophosphorus pesticides, parabens, alkylphenols, phenylphenols, BPA, TCS	Dairy products	Spain	LLE, SPE, derivatization (BSTFA + 1% TMCS)	GC-MS	LOD: 6–40 ng/kg RSD: 2.1–7.6% R: 80–108%	Parabens: 89–4300 ng/kg Phenylphenols: 130–3500 ng/kg BPA: 180–4800 ng/kg	This work

^a^ BPA: bisphenol A, BSTFA: N,O-bis(trimethylsilyl)trifluoroacetamide), DAD: diode array detector, DLLME: dispersive liquid-liquid microextraction, DMSPE: dispersive micro-solid phase extraction, DSPE: dispersive solid-phase microextraction, FLD: fluorescence detector, CG-MS: gas chromatography coupled to mass spectrometry, CG-MS/MS: gas chromatography coupled to tandem mass spectrometry, HPLC: high performance liquid chromatography, HPLC-MS/MS: high performance liquid chromatography coupled to tandem mass spectrometry, LLE: liquid–liquid extraction, LOD: limit of detection, LOQ: limit of quantification, ND: not detected, NMNTRA: N-mehtyl-N(trimethylsilyl)-trifluoroacetamide, NPD: nitrogen-phosphorus detector, QuEChERS: quick, easy, cheap, effective, robust and safe, R: recovery, SPE: solid-phase extraction, SPME: solid-phase microextraction, TCS: triclosan, TMCS: trimethylchlorosilane, UV: ultraviolet-visible detector.
